# Book Reviews

**Published:** 1896-05

**Authors:** 


### BOOK REVIEWS.

TEXT-BOOK UPON THE PATHOGENIC BACTERIA. For Students and Physicians. By Joseph McFarland, M.D., Demonstrator of Pathological Histology, and Lecturer on Bacteriology in the Medical Department of the University of Pennsylvania, etc. One hundred and thirteen illustrations. Price $2.50. W. B. Saunders, 925 Walnut street, Philadelphia.

This book is "intended to convey to the reader a concise account of the technical procedures necessary for the study of bacteriology, a brief description of the life-history of the important pathogenic bacteria, and sufficient description of the pathological lesions accompanying the invasions of micro-organisms to give an idea of the origin of symptoms and the causes of death" (preface).

A good introductory chapter places the reader in touch with the history of the study of bacteria, and of the germ theory of disease. Succeeding chapters discuss the biology of bacteria, the preparation of cultures and culture-media, the methods of observing and cultivating bacteria, sterilizing and disinfection, etc. The second part of the book discusses at length the inflammatory, toxic, and septic diseases in their relations with the respective micro-organisms producing them; such as, tuberculosis, leprosy, actinomycosis, diphtheria, tetanus, anthrax, typhoid fever, pneumonia, influenza, measles, relapsing fever, etc.

The book is complete, well written, well illustrated, and well printed. It will answer every requirement of a useful and practical work on this subject.

---

DAME FORTUNE SMILED. The Doctor's Story. Cloth, $1.25; paper, 50 cents. The Arena Publishing Company, Copley square, Boston, Mass.

This story is a retrospect from the year 1900. The narrative is simple and makes no pretence of literary excellence. The points, however, attract and hold the attention. The author teaches that the intense life of those who organize and carry through great speculative ventures is very exhaustive and provocative of nervous prostration; that when a man has acquired a fortune he should retire from "the Street" and devote at least a portion of his time and of his wealth to the betterment of the community in which he lives; that the pleasure experienced in giving and the hearty expressions of gratitude from the recipients of his benefactions will have a tendency to promote health and so prolong life. Incidental mention is made of the use of mental suggestion as a therapeutic agent, and running through the story are valuable medical suggestions in regard to preventive medicine for those who work under great nervous strain.

---

DISEASES OF THE RECTUM AND ANUS. Diagnosis and Treatment. For Practitioners and Students. By S. G. Gant, M.D., Professor of Diseases of the Rectum and Anus, University and Woman's Medical Colleges, Kansas City, Mo.; Surgeon to the German and to the Children's Hospital, etc. Sixteen full-page chromo-lithograph plates and 115 wood engravings. Published by the F. A. Davis Company, 1914 Cherry street, Philadelphia.

This treatise, we are told, is the result of an effort to give to practitioners and students a concise, yet practical work. The subject has been well covered, and all rectal disorders fully discussed from the standpoints of diagnosis and treatment. The author does not go into pathological minutiæ, though the important and essential facts of rectal pathology are by no means neglected. A very interesting portion of the book is the chapter on "Cancer" and "Colotomy," written by Mr. Herbert W. Allingham, of St. Mark's Hospital, London, whose large experience with this particular kind of cases makes him eminently qualified for the task which he assumed. We think practitioners will be highly pleased with this book.

---

ELECTRICITY IN ELECTRO-THERAPEUTICS. By E. J. Houston, Ph.D., and A. E. Kennelly, Sc.D. Published by The J. W. Johnston Company, 253 Broadway, New York. Price $1.00.

This book will give the reader some assistance in the principles of electricity, and in the many kinds of electrical apparatus which of course underlie the application of electricity to its many uses, including even therapeutics. But electro-therapeutics proper is not discussed, nor indeed are we told when and for what electricity may be used in the treatment of disease. However it is a very good little work on the fundamental principles of electricity.

---

DIETS FOR INFANTS AND CHILDREN. In Health and in Disease. By Louis Starr, M.D., Editor "American Text-Book of Diseases of Children." $1.25. W. B. Saunders, 925 Walnut street, Philadelphia.

This little book will serve a good purpose in suggesting to the physician in care of artificially-fed infants a suitable diet for different ages and for different conditions of health and disease. It is so arranged that leaves may be removed and given to mothers and nurses. There are also some good directions for the preparation of foods for children.

---

LIPPINCOTT'S MAGAZINE FOR MAY, 1896.

The complete novel in the May issue of *Lippincott's* is "An Impending Sword," by Horace Annesley Vachell. The hero enters the service of a California millionaire, whose domestic peace is endangered by the threats and wiles of a relentless enemy. Some queer entanglements ensue.

"Resaca," by Maurice Thompson, is a Georgia story of war-times. Carrie Blake Morgan turns from verse to prose to relate a cat's escape "From the Valley o' the Shadder." In "Love in the Afternoon," Clara E. Laughlin tells the successful matrimonial adventure of an elderly bachelor. What "The Fiddle Told," through the pen of Nora C. Franklin, was a story that abridged a life-term of imprisonment.

The tale of "The Last Duels in America" is told in detail by one of the principals in both, William Cecil Elam, now an editor in Norfolk. He tells it without rancor or bravado, and incidentally throws light on a state of society and public opinion now—in that respect at least—happily of the past.

Isabel F. Hapgood writes, out of abundant experience, on "Bed and Board in Russia." The need of "Official Residences for American Diplomats" is pointed out by Theodore Stanton. "Highways of the Sea and the Steamships which Traverse Them" are described by Clarence H. New.

"The Abbey of Gethsemane," to which Allen Hendricks conducts the reader, is the Trappist monastery in Kentucky. "An Overlooked Poet" of a hundred years ago is brought to light by F. M. B. The poetry of the number is by Hattie Whitney, Ida Whipple Benham, Madison Cawein, and Clarence Urmy.

